# Immunity elicitors for induced resistance against the downy mildew pathogen in pearl millet

**DOI:** 10.1038/s41598-022-07839-4

**Published:** 2022-03-08

**Authors:** Senapathyhally Nagaraju Lavanya, Sathyanarayana Niranjan-Raj, Ragi Jadimurthy, Sujesh Sudarsan, Rakesh Srivastava, C. Tarasatyavati, H. Rajashekara, Vijai Kumar Gupta, Siddaiah Chandra Nayaka

**Affiliations:** 1 Department of Studies in Biotechnology, University of Mysore, Manasagangotri, Mysuru, Karnataka India; 2 Department of Studies in Microbiology, Karnataka State Open University, Mukthagangotri, Mysuru, Karnataka India; 3 Department of Studies in Molecular Biology, University of Mysore, Manasagangotri, Mysuru, Karnataka India; 4 International Crops Research Institute for the Semi-Arid Tropics (ICRISAT), Patancheru, Telangana 502324 India; 5 All India Coordinated Research Project on Pearl Millet, Indian Council of Agricultural Research, Mandor, Jodhpur, Rajasthan 342304 India; 6 Crop Protection Section, ICAR-Directorate of Cashew Research (DCR), Dakshina Kannada, Puttur, Karnataka 574202 India; 7 Biorefining and Advanced Materials Research Center, Scotland’s Rural College (SRUC),, Kings Buildings, West Mains Road, Edinburgh, EH9 3JG UK; 8 Center for Safe and Improved Food, Scotland’s Rural College (SRUC), Kings Buildings, West Mains Road, Edinburgh, EH9 3JG UK

**Keywords:** Biochemistry, Biotechnology, Microbiology, Plant sciences

## Abstract

Pearl millet (*Pennisetum glaucum* (L.) R. Br.) is a globally important cereal whose production is severely constrained by downy mildew caused by *Sclerospora graminicola* (Sacc.). In this study, immunity eliciting properties of 3,5-dichloroanthranilic acid (DCA), Cell Wall Glucan (CWG), Lipopolysaccharide (LPS), and Glycinebetaine (GB) was deciphered through enzymatic and protein studies based on elicitor treatment activated defense mechanisms. Glycinebetaine, LPS, CWS and DCA elicited enzyme activities and gene expression of the defense enzymes, such as β-1,3-glucanase, phenylalanine ammonia lyase (PAL), peroxidase (POX), polyphenol oxidase (PPO), lipoxygenase (LOX) and defense protein hydroxyproline-rich glycoproteins (HRGPs). However, the speed and the extent of elicitation differed. High levels of enzyme activities and gene expression in elicitor-treated *P. glaucum* positively correlated with the increased downy mildew resistance. A very rapid and large changes in elicitor-treated seedlings, in contrast to the delayed, smaller changes in the untreated susceptible control seedlings suggests that the rate and magnitude of defense gene expression are important for effective manifestation of defense against pathogen. As compared to other elicitors and control, GB promoted increase in enzyme activities and gene expression, implicating that GB is a promising elicitor of downy mildew resistance in *P. glaucum*.

## Introduction

Host defense responses begin at the pathogen attack site and subsequently extend to the surrounding cells, thereby systemically activating the resistance signal and further manifesting the defense responses throughout the plant, leading to systemic acquired resistance (SAR). Induced systemic resistance (ISR) is based on multiple mechanisms, including enhancement of the plants’ ability to activate cellular defense responses upon pathogen challenge^1,2^.

Phenolic compounds in the plant metabolic pool are greatly influenced by the treatment with different elicitors, which eventually result in enhanced phenylalanine ammonia lyase (PAL), peroxidase (POX) and polyphenoloxidase (PPO) activities^3,4^. Phenylalanine ammonia lyase is an important enzyme of the phenylpropanoid pathway involved in the biosynthesis of phenolic compounds and the signaling molecule salicylic acid (SA)^5^. Peroxidases have a variety of roles, including hypersensitive response (HR), lignification, phenolic and glycoprotein cross-linking, suberization, and phytoalexin synthesis^6,7^. By oxidizing phenolic chemicals to harmful antimicrobial quinones, the PPO enzyme plays a vital role in defense mechanisms^8^. It is also thought to play a function in lignin production. Plant defense proteins such as β-1,3-glucanase and chitinases are extensively studied and known to be involved in plant resistance to fungal infections^9^. Induced by pathogen infection or inducer treatment, these enzymes suppress fungal growth and release oligosaccharide elicitors, resulting in the generation of phytoalexins^10,11^. Lipoxygenase attacks the plant membrane system non-enzymatically and stimulates lipid peroxidation, which controls defense responses such as tissue necrosis, H_2_O_2_ buildup, and HR. Hydroxyproline-rich glycoproteins (HRGPs) are the structural components of plant cell walls that play an essential role in host defense responses^12^. Infections with pathogens or treatments with pathogen-derived elicitors raise HRGP levels, which leads to resistance to a variety of diseases^13–15^.

Numerous investigations have established that during elicitor-induced systemic resistance to a broad range of pathogens, defense enzymes like PAL, POX, PPO, β-1,3-glucanase, chitinase, LOX, catalase and others are significantly over-expressed and early in expression compared to control^16–19^. *Pseudomonas fluorescens*, *Trichoderma viride,* and *Trichoderma harzianum* are applied as soil amendments to coconut plantation, induced resistance against *Ganoderma* disease caused by *Ganoderma lucidum* and also showed enhanced levels of POX, PAL, PPO, glucanase, and chitinase^20^. Cell-wall protein (CWP) of *Fusarium oxysporum* f. sp. *ciceri* and *Macrophomina phaseolina* induced systemic resistance against *Fusarium* wilt and charcoal rot in chickpea, respectively, and it was accompanied by increased activities of PAL and POX enzymes, pathogenesis-related (PR) proteins, and phenolics^21^. Tomato plants when treated with hormonal elicitors SA and indole acetic acid showed enhanced resistance against *Orobanche ramose* infection and also enhanced activities of the defense enzymes PAL, POX, and PPO^22^. Garampalli et al.^23^ reported significantly increased levels of different enzymes like PAL and POX with inoculations of different plant extracts like *Duranta repens, Polyalthia longifolia,* and *Parthenium hysterophorus,* which led to systemic disease protection against sorghum downy mildew. Treatment with β- Aminobutyric Acid (BABA) stimulated defense responses in squash plants, and resulted in resistance to powdery mildew fungus, which correlated with the increased expression of PR-1, PAL and POX^24^ genes . Spray treatment of pea plants with different elicitors such as SA, *P. fluorescens*, Chitosan, and *T. harzianum* induced higher levels of phenols, POX, PAL, and PPO leading to resistance against the rust pathogen *Uromyces viciae–fabae* (Pers.) J. Schrot^25^.

The role of defense enzymes such as PAL, POX, PPO, glucanase, RNase, proton ATPase, superoxide dismutase (SOD), LOX, and defense protein HRGPs in *P. glaucum* resistance against downy mildew has been shown in earlier studies and these defense enzymes and proteins act as markers of *P. glaucum* downy mildew resistance^26–35^. Therefore, the main objective of the present study was to monitor some of the metabolic changes due to different elicitors treatment, and elucidate the role of various defense enzymes like glucanase, PAL, POX, PPO, LOX and HRGPs and how their activities influence the induction and development of resistance in *P. glaucum*.

## Materials and methods

### Plant materials

Seeds of *P. glaucum* 7042S (Susceptible cultivar) and IP 18292 (Resistant cultivar) to downy mildew were obtained from All India Coordinated Research Project on Pearl Millet (AICRP-PM), Mandore, Rajasthan, India and International Crops Research Institute for the Semi-Arid Tropics (ICRISAT), Patancheru, Hyderabad, Andhra Pradesh, India. Authors confirm that the plants used in the present study complies with international, national and/or institutional guidelines.

### Pathogen source and inoculum preparation

Throughout the investigation, downy mildew infected samples were collected from extensively diseased leaves of 21-day old seedlings of the susceptible cultivar (7042S) cultivated in the downy mildew sick plot (Mysore). Infected *P. glaucum* leaves with abundant sporulation were collected in the evening, rinsed well in running tap water to remove existing sporangia, blot dried, and stored overnight for sporulation. After 24 h of incubation, fresh sporangia were harvested. The spore load was adjusted to 40,000 zoospores/ml using a Haemocytometer and used as inoculum for all further studies.

### Preparation of elicitors and seed treatment

#### Cell wall glucan (CWG)

The endophytic strain of *Trichoderma hamatum* UOM 13 (accession no. KP876050) which had host resistance inducing potential was isolated from *P. glaucum* rhizosphere, and identified based on the microscopic and macroscopic characteristics. Amplification and sequencing a portion of the internal transcribed spacer (ITS) region further validated it at the molecular level^36,37^. The CWG elicitors were extracted from *T. hamatum* UOM 13 strain by adopting the procedure described by Sriram et al.^38^.

#### Lipopolysaccharide (LPS)

The plant growth-promoting rhizobacteria *Pseudomonas fluorescens* UOM SAR 14 (Biosample Accession No. SAMN06286563) was isolated from the *P. glaucum* rhizosphere and maintained on King’s Medium B (KMB). The bacterial suspension was prepared by scraping colonies grown overnight from KMB agar plates in 0.01 M magnesium sulphate. The isolation and preparation of LPS from the bacterial cell suspensions were essentially carried out as described by Lavanya et al*.*^39^.

#### Glycinebetaine (GB)

Glycinebetaine was obtained from Sisco Research Laboratories Pvt. Ltd. Mumbai, and GB solutions were prepared at different concentrations of 10, 20, 30, 40 and 50 mg/mL in sterile distilled water, and mixed on a stirrer for a few minutes to ensure complete solubilization till no granules were left over.

#### 3,5-dichloroanthranilic acid (DCA)

3,5-dichloroanthranilic acid was obtained from Sigma, Germany, andDCA solutions were prepared by dissolving 10, 20, 50, 100 µM in DMSO (1 µl/mL) of sterile distilled water, and kept for constant agitation for 2 h for complete dissolution.

### Seed treatment with different elicitors

The seeds of 7042S were surface sterilized for 5 min with 0.02% mercuric chloride and thoroughly rinsed in sterile distilled water. Seeds were treated by soaking them (5 g/25 mL) in the elicitor solutions of various concentrations, as stated above, and then adding 0.2% sterilized carboxymethyl cellulose (CMC) as an adhesive substance. The suspensions were incubated at 26 °C in a rotary shaker for 2, 4 and 6 h to facilitate uniform attachment of the elicitors to seed coat. Seeds treated with distilled water and amended with CMC served as untreated control. The concentration and treatment time interval, which did not inhibit the seed germination and seedling vigor were used for further experimental studies.

### Plating of treated seeds

The 7042S cultivar seed treatments with various elicitors were the same as previously described. The IP 18292 seeds treated with distilled water were also used as a resistant control. Treated seeds were plated on pre-soaked blotters in Perspex plates and incubated for 2 days.

### Challenge inoculation and harvesting of seedlings

Two-days-old seedlings (7042S and IP 18292) were root-dip infected with a zoospore suspension containing 40,000 zoospores/ml and incubated in dark at 25 ± 2 °C. One set of the treated seedlings inoculated with sterile distilled water, served as un-inoculated control. For each experiment, 1 g seedlings were taken at 0, 3, 6, 9, 12, 24, 48, and 72 h after inoculation (hpi) in three replicates, immediately wrapped in aluminum foil and stored at − 80 °C until further use^40^.

### Extraction of samples for enzyme and protein assays

The harvested seedlings (1 g fresh weight) were washed thoroughly in sterile distilled water, homogenized with liquid nitrogen, ground to a fine paste with 1 mL of respective buffers with a mortar and pestle, and filtered through a 0.20 mm nylon filter into a centrifuge tube. The seedlings extracts were centrifuged at 12,000*g* for 20 min at 4 °C. The supernatant was transferred to a 1.5 mL vial, and stored at − 20 °C till further use for biochemical assays. A colorimetric assay for enzymatic activity was performed with Hitachi 2000 Spectrophotometer. The reaction rates were linear and proportional to the enzyme or protein concentration added.

### Protein estimation

Protein content in the extracts was estimated using the protein dye-binding method^41^ using Bovine Serum Albumin (BSA) (Sigma, USA) as a standard.

### Phenylalanine ammonia-lyase assay (PAL, EC. 4.1.3.5)

A 25 mM Tris HCl buffer (pH 8.8) was used to extract the PAL enzyme. A 100 µl of plant extract was mixed with 900 ml of 50 mM l-Phenylalanine and 100 mM Tris HCl buffer solution (pH 8.01). The mixture was kept in a water bath at 40 °C for 120 min. The reaction was halted by adding 60 ml of 5 N HCl. The PAL activity was measured as described by Beaudoin-Eagan and Thorpe^42^. The enzyme activity was expressed in terms of µmol tcinnamic acid mg^−1^ protein min^−1^.

### Peroxidase assay (POX, EC.1.11.1.7)

The POX enzyme was extracted in a 10 mM potassium phosphate buffer (pH 6.9), and its activity was measured as described by Hammerschmidt et al*.*^43^. The reaction mixture consisted of (3 mL). 0.25% (v/v) guaiacol in 10 mM potassium phosphate buffer (pH 6.0) and 100 mM hydrogen peroxidase The reaction was started using 10 ml of crude enzyme, and detected spectrophotometrically at 470 nm. The POX activity is expressed in terms of the change in A_470_ min^−1^ mg^−1^ protein.

### β-1,3-Glucanase assay (GLU, EC. 3.2.1.6)

β-1,3-Glucanase enzyme was extracted in a 50 mM sodium acetate buffer (pH 5.2). Its activity was assayed as described by Pan et al*. *with glucose as standard^44^. Reaction mixture consisting Laminarin (0.1%, Sigma) in 0.05 M sodium acetate buffer (pH 5.2) and 50 ml enzyme extract was incubated for 15 min at 37 °C. The reaction was halted by adding 0.5 ml of dinitro salicylate reagent, incubated in boiling water bath for 10 min, cooled, followed by addition of 1 ml distilled water. Products released after incubation were estimated for reducing groups (d-glucose) at 540 nm. The enzyme activity was expressed in terms of µmoles mg^−1^ min^−1^.

### Polyphenol oxidase assay (PPO, EC. 1.14.18.1)

Tris–HCl buffer (pH 7.0) containing 0.1 M KCl, 1% (v/v) Triton X-100, 1 mM EDTA and 5% (w/v) polyvinylpolypyrrolidone (PVPP) was used to extract polyphenoloxidase. The standard reaction mixture consisted of 3 ml of 10 mM sublimated catechol in 100 mM potassium phosphate buffer (pH 6.5), and 10 ml of enzyme extract as described by Niranjan Raj et al.^29^. An increase in absorbance at 420 nm was recorded for 1 min. The results were expressed as the change in absorbance min^−1^ mg^−1^ protein.

### Lipoxygenase assay (LOX, EC. 1.13.11.12)

Lipoxygenase enzyme was extracted with 0.2 M sodium phosphate buffer (pH 6.5). The LOX activity was measured as per the procedure of Borthakur et al.^45^. The activity was determined spectrophotometrically by monitoring the appearance of the conjugated diene hydroperoxide at 234 nm. The substrate for the LOX assay was prepared according to the method described by Axelrod et al.^46^. Linoleic acid (70 µl) was mixed with an equal volume of Tween-20 plus and 3 ml of distilled water. A 125 ml of 2 N NaOH was added to obtain a clear solution. The volume of the solution was made up to 25 ml with distilled water. Each time, the substrate was prepared fresh and used for the enzyme assay. The reaction mixture contained 2.7 ml of sodium phosphate buffer (0.2 M, pH 6.5) and 0.3 ml of the substrate. The reaction was initiated by adding the 10 ml enzyme extract and the change in absorbance at 234 nm was recorded. The enzyme activity was expressed as a change in the absorbance (Δ_234_) mg^−1^ protein min^−1^.

### Analysis of hydroxyproline-rich glycoproteins (HRGPs)

Cell walls from seedlings of *P. glaucum* were obtained by modifying the established procedure of York et al.^47^. The seedlings were homogenized using pestle and mortar at 4 °C in 0.5 M potassium phosphate buffer (pH 7.0).The paste was examined under a microscope for complete cell rupture. The broken cell suspension was centrifuged for 10 min at 2000*g*. The above buffer was used to wash the cell walls several times before rinsing with distilled water. The washed cell walls were suspended in 5 L of 1:1 chloroform–methanol by vigorous swirling. The cell walls were washed repeatedly in 5 volumes of acetone and dried in the open air. The amount of HRGPs was determined by analyzing the hydroxyproline (Hyp) content in the cell wall hydrolysate.

In sealed tubes, cell walls were hydrolyzed in 6 N HCl for 18 h at 110 °C. The hydrolysates were evaporated to dryness to eliminate the HCl. Hydroxyproline was then extracted in a small amount of sterile distilled water from the dried hydrolyzed samples and determined following the spectrophotometric method of Prockop and Udenfriend^48^. Hydroxyprolinecontent was expressed as µg Hyp mg^−1^cell wall (dry weight).

### Quantitative real-time PCR (qPCR) analysis

#### RNA extraction

A total of 100 mg of frozen seedlings were ground to a fine powder in 2 ml SealRite microcentrifuge tube using stainless steel beads and an automated shaker SO-10 M (Fluid Management, Wheeling, IL, USA). Total RNA was extracted from seedlings harvested at different times noted above by using RNeasy plant mini Kit (Qiagen) as per the manufacturer’s instructions. The eluted RNA was stored at − 80 °C and treated with DNase I (RNase free) (Fermentas). The concentration, purity and integrity of RNA was determined spectrophotometrically using Nanodrop ND-1000 (Thermo Scientific) and agarose gel (1.2%) electrophoresis.

#### qRT-PCR analysis

The relative quantification of PAL (NM001174615.1), POX (EU492461), PPO (AY881993.1), β-1,3-glucanase (EU725041.1), LOX (AF329371.1), and HRGP (GQ223398) *mRNAs* in *P. glaucum* seedlings was done by using gene-specific primers^49^, designed with Primer Express version 3.0 software (Applied Biosystems) (Supplementary Table 1). Primers for PPO, β-1,3-glucanase, LOX and HRGP were designed, and primers for PAL and POX, were selected from Nayak et al*.*^37^. Protein phosphatase 2A (PP2A ) served as endogenous reference control. Primer specificities were confirmed by agarose gel electrophoresis of the RT-PCR products. Each qPCR reaction (20 μL) consisted of 1× SYBR Green PCR master mix (SYBR Green mix, Applied Biosystems), 3 pmol of each primer and 20 ng cDNA. StepOnePlus™ Real-Time PCR Systems (Applied Biosystems) was used for denaturation at 95 °C for 10 min, 40 cycles of 15 s at 95 °C, and 60 s at 60 °C. The relative expression levels were determined by using comparative threshold method^50^.

### Statistical analysis

All the biochemical and molecular experiments were performed in four replicates. Data were analyzed separately for each experiment and was subjected to arcsine transformation. The analysis of variance was carried out with transformation values (JMP Software; SAS Institute Inc., Cary, NC). The significance of the effect of treatments was determined by the magnitude of the F value (*P* ≤ 0.05). Treatment means were separated by Tukey’s HSD test.

## Results

### Phenylalanine ammonia lyase (PAL) activity

**Figure 1:**
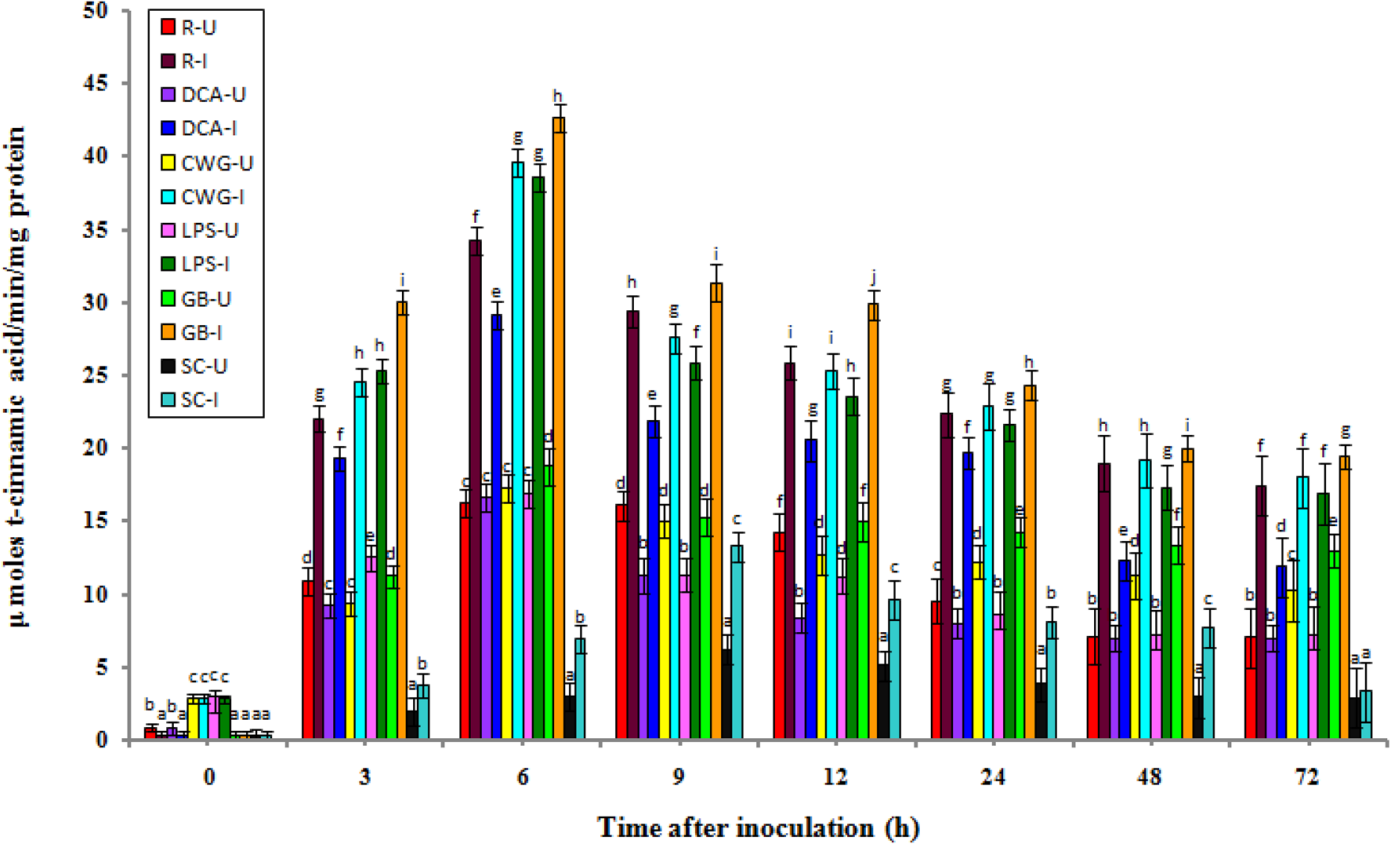
Temporal pattern of PAL activity in 2-day old *P. glaucum* seedlings with (I-inoculated) or without (U-uninoculated) *Sclerospora graminicola* inoculation. R—resistant, S—susceptible, DCA—3,5-Dichloroanthranilic acid treated, CWG—Cell Wall Glucans isolated from the endophyte *Trichoderma hamatum* UOM 13, LPS—Lipopolysaccharides isolated from bacteria *Pseudomonas fluorescens* UOM 14, GB-Glycinebetaine an amino acid derivative. Bars indicate standard errors; means with different superscripts are significantly different, as shown by Tukey’s HSD test (*p* ≤ 0.05).

In general, PAL activity levels were recorded in all categories of seedlings of with or without treatment). In both treatment samples, Phenylalanine ammonia lyase activity was gradually increased after 3 h of incubation and its peak was obtained after 6 h in the elicitor treated samples and resistant seedlings. In contrast, PAL activity peaked at 9 h in susceptible seedlings. Inoculated seedlings had considerably higher PAL activity than uninoculated seedlings in all treatments and at all time periods assessed (Fig. 1).

In pathogen-inoculated seedlings, PAL activity peaked at 6 hpi in resistant and elicitor treated seedlings, while in susceptible control seedlings PAL activity peaked at 9 hpi. The maximum PAL activity was noted in GB-treated seedlings, which were even significantly higher than resistant seedlings. Among the elicitor treatments, at 6 hpi, GB-treated seedlings recorded 42.66 units PAL activity, followed by CWG, LPS, and DCA treatments, which recorded 39.6, 38.56, and 29.15 units PAL activity, respectively, whereas in the control seedlings it showed 6.92 units PAL activity. Phenylalanine ammonia lyase activity in GB, CWS, LPS, and DCA treated seedlings was 6.16-, 5.72-, 5.57-, and 4.21-folds higher at 6 hpi than that of the control, respectively. Resistant seedlings showed 34.26 units PAL activity and it was 4.95-folds higher than the control. At 6 hpi GB-treated seedlings showed 1.07-, 1.11-, and 1.46-fold higher PAL activity than CWG, LPS, and DCA treatments, respectively.

In seedlings without pathogen inoculation, the pattern of PAL activity was similar to that of pathogen inoculated seedling but the level of activity was significantly lesser. Among the elicitor treatments at 6 h, GB-treated seedlings recorded maximum PAL activity by showing 18.75 activity followed by CWG, LPS, and DCA treatments which showed 17.28, 16.92, and 16.61 units PAL activity, respectively compared to control seedlings which recorded 2.96 PAL activity. The GB-treated seedlings showed 6.33-folds higher activity than the control at 6 h.

At 6 h PAL activity in challenge inoculation in treated seedlings of GB, CWG, LPS, and DCA, results showed 2.27-, 2.29-, 2.27-, and 1.75-folds higher than that of the un-inoculated samples, respectively.

### Peroxidase (POX) activity

**Figure 2:**
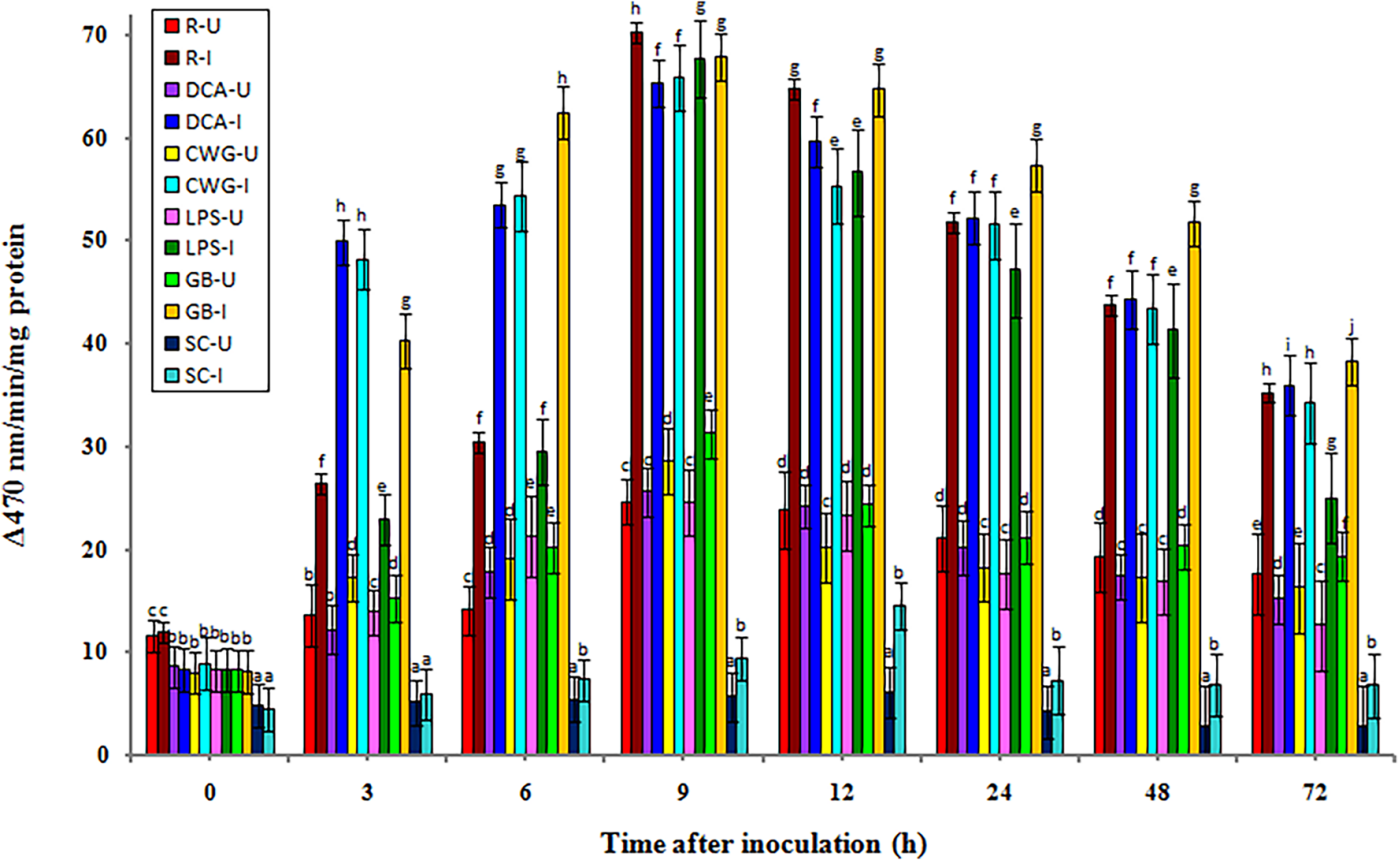
Temporal pattern of POX activity in 2-day old *P. glaucum* seedlings with (I-inoculated) or without (U-uninoculated) *Sclerospora graminicola* inoculation. R-resistant, S-susceptible, DCA-3,5-Dichloroanthranilic acid treated, CWG—Cell Wall Glucans isolated from the endophyte *Trichoderma hamatum* UOM 13, LPS—Lipopolysaccharides isolated from bacteria *Pseudomonas fluorescens* UOM 14, GB—Glycinebetaine an amino acid derivative. Bars indicate standard errors; means with different superscripts are significantly different, as shown by Tukey’s HSD test (*p* ≤ 0.05).

The constitutive POX activity was recorded in all categories of seedlings in both treatments (with or without pathogen inoculation). In both pathogen inoculated and uninoculated samples, POX enzyme activity gradually increased from 3 h onwards and peaked at 9 h in samples of elicitor treated and resistant seedlings, whereas in susceptible seedlings POX activity peaked at 12 h. The POX enzymatic activity was significantly higher in inoculated seedlings compared to the uninoculated seedlings in all treatments and at all tested time intervals (Fig. 2).

In pathogen-inoculated seedlings the POX activity was peaked at 9 hpi in both resistant and elicitor treated seedlings whereas in susceptible control seedlings, its activity peaked at 12 hpi. The maximum POX enzymatic activity was observed in resistant seedlings, which recorded 70.28, units and it was 7.47-folds higher than the susceptible control seedlings. Among the elicitor treated samples, at 9 hpi, GB and LPS treated seedlings recorded 67.86 and 67.73 units POX activity, respectively which were not statistically significant from each other, followed by CWG and DCA treatments which recorded 65.9 and 65.33 POX activity, respectively, whereas in control seedlings it showed 9.4 units POX activities. At 9 hpi, POX activity in GB, LPS, CWS, and DCA treated seedlings was 7.22-, 7.20-, 7.01-, and 6.95-folds higher than control seedlings, respectively.

In seedlings without pathogen inoculation, the pattern of POX activity was similar to pathogen inoculated seedling, but the activity level was significantly lesser. Among the elicitor treatments at 9 h, GB-treated seedlings recorded maximum POX activity by showing 31.32 units activity followed by, CWG, DCA, LPS, and treatments which showed 28.65, 25.58, and 24.54 POX activity, respectively, compared to the control seedlings with 5.71 units POX activity. GB treatment showed 5.48-folds higher activity than the susceptible control at 9 h.

At 9 h POX activity in pathogen inoculated GB, LPS, CWG, and DCA treated seedlings was 2.16-, 2.75-, 2.3-, and 2.55-folds higher than that of the uninoculated samples, respectively.

### Polyphenoloxidase (PPO) activity

**Figure 3:**
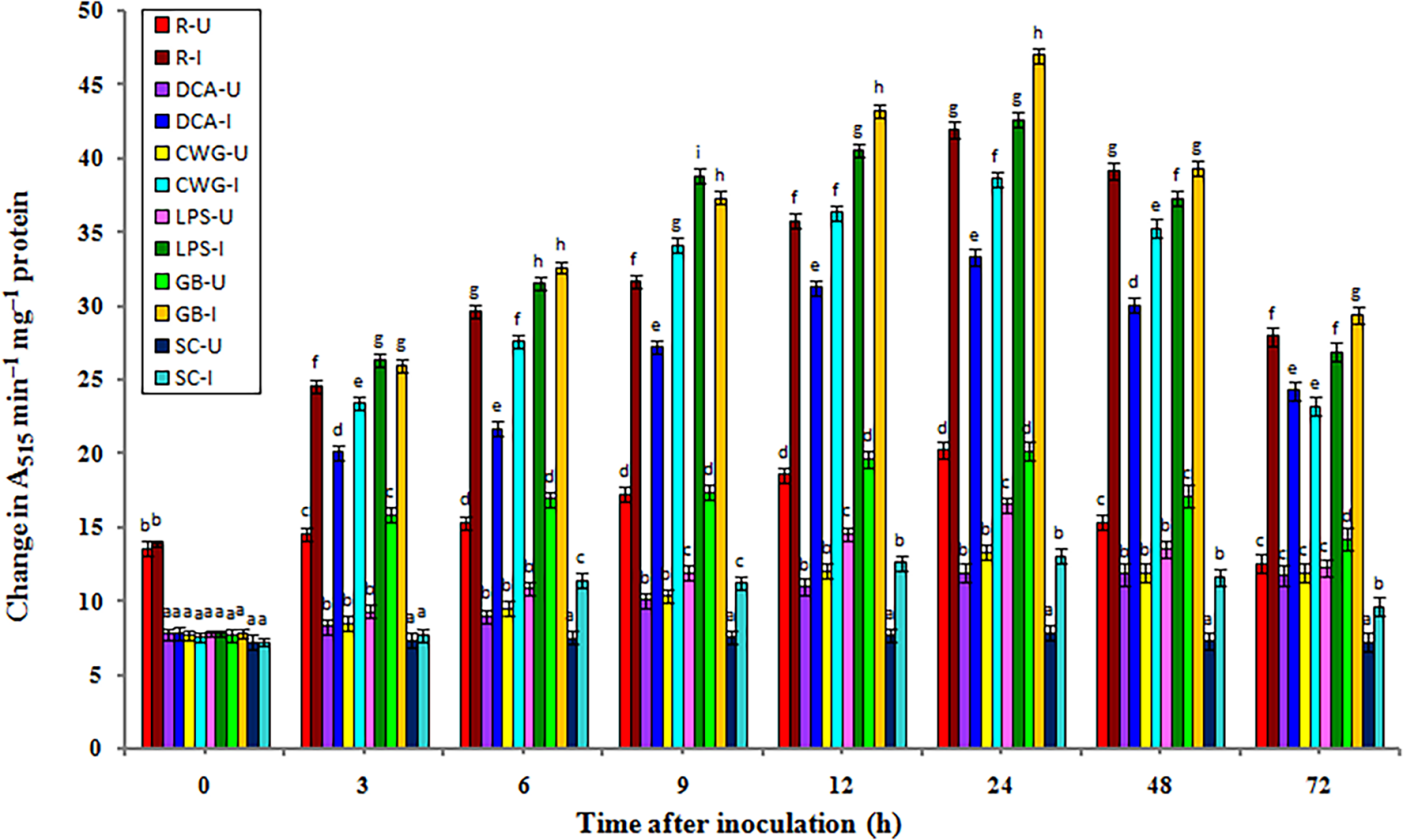
Temporal pattern of PPO activity in 2-day old *P. glaucum* seedlings with (I-inoculated) or without (U-uninoculated) *Sclerospora graminicola* inoculation. R—resistant, S—susceptible, DCA—3,5-Dichloroanthranilic acid treated, CWG—Cell Wall Glucans isolated from the endophyte *Trichoderma hamatum* UOM 13, LPS—Lipopolysaccharides isolated from bacteria *Pseudomonas fluorescens* UOM 14, GB—Glycinebetaine an amino acid derivative. Bars indicate standard errors; means with different superscripts are significantly different, as shown by Tukey’s HSD test (*p* ≤ 0.05).

In general, the constitutive PPO activity was recorded in all categories of seedlings with or without pathogen inoculation. In both pathogen inoculated and uninoculated samples PPO activity gradually increased from 3 h onwards and peaked at 24 h in elicitor treated, resistant, and control seedlings. Polyphenoloxidase activity was significantly higher in inoculated seedlings compared to the uninoculated seedlings in all treatments and at all tested time intervals (Fig. 3).

In pathogen inoculated seedlings, PPO activity peaked at 24 hpi, and maximum PPO activity was observed in GB-treated seedlings, which was even significantly higher than resistant seedlings. Among the elicitor treatments, at 24 hpi, GB-treated seedlings recorded 47.02 units PPO activity followed by LPS, CWG, and DCA treatments which recorded 42.66, 38.65, and 33.32 units PPO activity, respectively, whereas the control seedlings showed 13.09 PPO activity. Polyphenoloxidaseactivity at 24 hpi h in GB, LPS, CWS, and DCA-treated seedlings was 3.59-, 3.25-, 2.95-, and 2.54-folds higher than that of the control seedlings, respectively. Resistant seedlings showed 41.95 units PPO activity which was 3.2-folds higher than the control seedlings. At 24 hpi GB-treated seedlings showed 1.1-, 1.21-, and 1.41-folds higher PPO activity than LPS, CWG, and DCA treatments, respectively.

In seedlings without pathogen inoculation, the pattern of PPO activity was similar to that of pathogen inoculated seedling but the level of activity was significantly lesser. Among the elicitor treatments at 24 h, GB-treated seedlings recorded maximum PPO activity by showing 20.18 units activity followed by LPS, CWG, and DCA treatments, which showed 16.58, 13.37, and 11.94 units PPO activity, respectively compared to the control seedlings which showed 7.87 PPO activity. GB treatment showed 2.56-folds higher activity than the susceptible control at 24 h.

At 24 h PPO activity in pathogen inoculated GB, LPS, CWG, and DCA treated seedlings was 2.33-, 2.57-, 2.89-, and 2.79-folds higher than that of the uninoculated samples, respectively.

### β-1,3-glucanase activity

**Figure 4:**
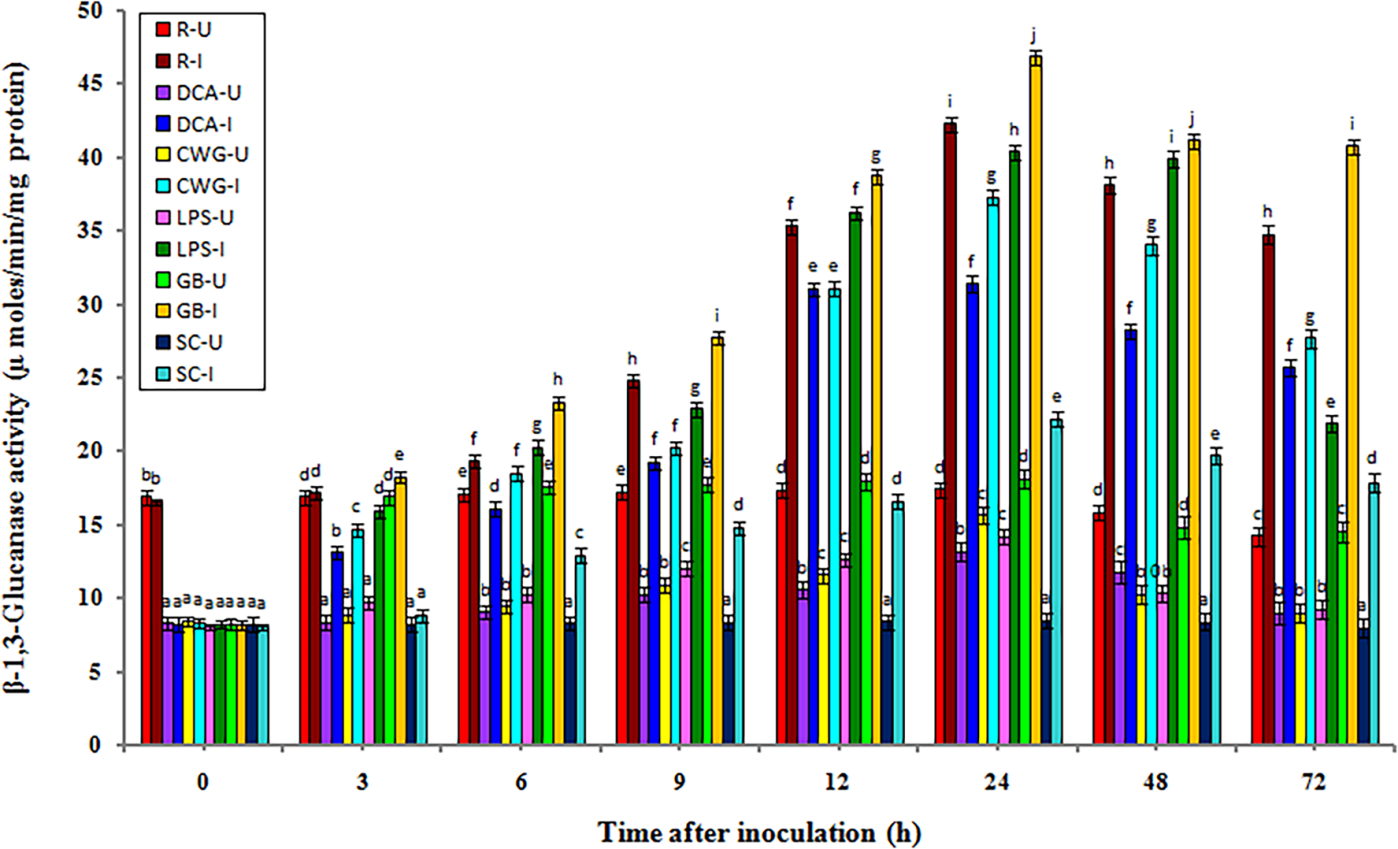
Temporal pattern of β-1,3-glucanase activity in 2-day old *P. glaucum* seedlings with (I-inoculated) or without (U-uninoculated) *Sclerospora graminicola* inoculation. R—resistant, S—susceptible, DCA—3,5-Dichloroanthranilic acid treated, CWG—Cell Wall Glucans isolated from the endophyte *Trichoderma hamatum* UOM 13, LPS—Lipopolysaccharides isolated from bacteria *Pseudomonas fluorescens* UOM 14, GB—Glycinebetaine an amino acid derivative. Bars indicate standard errors; means with different superscripts are significantly different, as shown by Tukey’s HSD test (*p* ≤ 0.05).

In general, constitutive levels of β-1,3-glucanase activity were recorded in all categories of seedlings with or without pathogen inoculation, however, the activities were significantly higher in resistant seedlings compared to the other categories of seedlings. In both pathogen inoculated and uninoculated samples β-1,3-glucanase activity gradually increased from 3 h onwards and peaked at 24 h in elicitor treated, resistant, and control seedlings. The β-1,3-glucanase activity was significantly higher in inoculated seedlings compared to the uninoculated seedlings in all treatments and at all tested time intervals (Fig. 4).

In pathogen inoculated seedlings, β-1,3-glucanase activity peaked at 24 hpi, and maximum activity was observed in GB-treated seedlings which was even significantly higher than resistant seedlings. Among the elicitor treatments, at 24 hpi, GB-treated seedlings recorded 46.86 units β-1,3-glucanase activity, followed by LPS, CWG, and DCA treatments which recorded 40.36, 37.26, and 31.36 units β-1,3-glucanase activity, respectively, whereas control seedlings showed 22.2 units -1,3-glucanase activity. At 24 hpi β-1,3-glucanase activity in GB, LPS, CWS, and DCA treated seedlings was 2.11-, 1.81-, 1.67-, and 1.41-folds higher than that of the control, respectively. Resistant seedlings showed 42.24 β-1,3-glucanase activity which was 1.9-folds higher than the control seedlings. At 24 hpi GB-treated seedlings showed 1.16, 1.25, and 1.49 folds higher β-1,3-glucanase activity than LPS, CWG, and DCA treatments, respectively.

In seedlings without pathogen inoculation, the pattern of β-1,3-glucanase activity was similar to that of pathogen inoculated seedling, but the activity level was significantly lesser. Among the elicitor treatments at 24 h, GB-treated seedlings recorded maximum β-1,3-glucanase activity by showing 18.16 units activity followed by CWG, LPS, and DCA treatments which showed 15.65, 14.18, and 13.15 units β-1,3-glucanase activity, respectively compared to the control seedlings which showed 8.45 β-1,3-glucanase activity. GB treatment showed 2.16-folds higher activity than the susceptible control at 24 h.

At 24 h β-1,3-glucanase activity in pathogen inoculated GB, LPS, CWG, and DCA treated seedlings was 2.58-, 2.84-, 2.38-, and 2.40-folds higher than that of the uninoculated samples, respectively.

### Lipoxygenase activity

**Figure 5:**
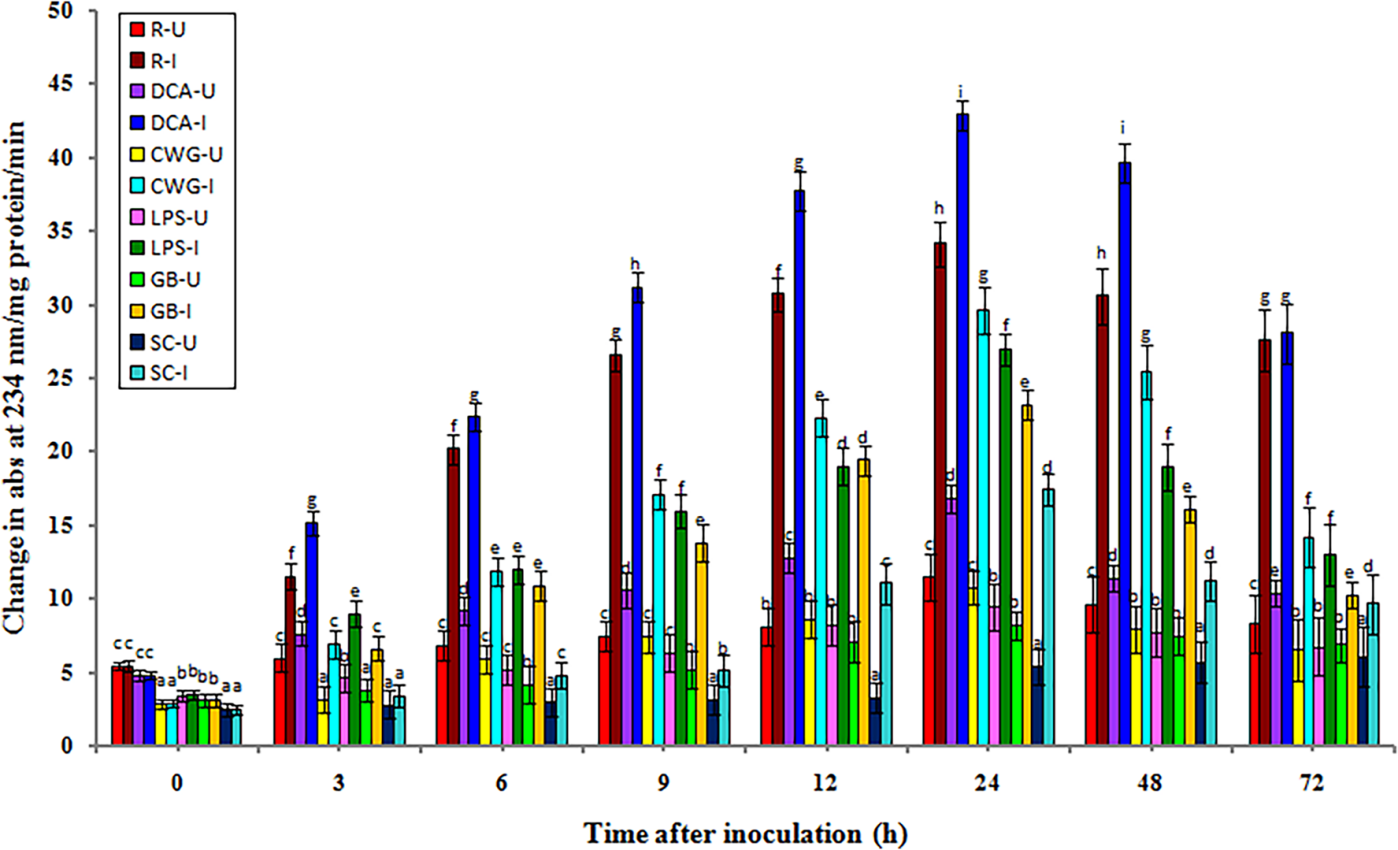
Temporal pattern of LOX activity in 2-day old *P. glaucum* seedlings with (I-inoculated) or without (U-uninoculated) *Sclerospora graminicola* inoculation. R—resistant, S—susceptible, DCA—3,5-Dichloroanthranilic acid treated, CWG—Cell Wall Glucans isolated from the endophyte *Trichoderma hamatum* UOM 13, LPS—Lipopolysaccharides isolated from bacteria *Pseudomonas fluorescens* UOM 14, GB—Glycinebetaine an amino acid derivative. Bars indicate standard errors; means with different superscripts are significantly different, as shown by Tukey’s HSD test (*p* ≤ 0.05).

In general, the constitutive level of LOX activity was recorded in all categories of seedlings with or without pathogen inoculation. In both pathogen inoculated and uninoculated samples LOX activity gradually increased from 3 h onwards and peaked at 24 h in elicitor treated, resistant, and control seedlings. LOX activity was significantly higher in inoculated seedlings compared to the uninoculated seedlings in all treatments and at all tested time intervals (Fig. 5).

In pathogen inoculated seedlings LOX activity peaked at 24 hpi and maximum LOX activity was observed in DCA treated seedlings, which was even significantly higher than resistant seedlings. Among the elicitor treatments, at 24 hpi, DCA-treated seedlings recorded 42.88 units LOX activity, followed by CWG, LPS, and GB treatments, which recorded 29.63, 27, and 23.19 LOX activity, respectively, whereas the control seedlings showed 17.42 units LOX activity. LOX activity at 24 hpi in DCA, CWG, LPS, and GB-treated seedlings was 2.4-, 1.7-, 1.55-, and 1.33-folds higher than that of the control seedlings, respectively. Resistant seedlings showed 34.18 LOX activity which was 1.98-folds higher than the control seedlings. At 24 hpi DCA treated seedlings showed 1.45-, 1.59-, and 1.85-fold higher LOX activity than CWG, LPS, and GB treatments, respectively.

In seedlings without pathogen inoculation, the pattern of LOX activity was similar to that of pathogen inoculated seedling but the level of activity was significantly lesser. Among the elicitor treatments at 24 h, DCA-treated seedlings recorded maximum LOX activity by showing 16.83 activity followed by CWG, LPS, and GB treatments which showed 10.8, 9.47, and 8.2 units LOX activity, respectively compared to the control seedlings which showed 5.4 LOX activity. DCA treatment showed 3.12-folds higher activity than the susceptible control at 24 h.

At 24 h LOX activity in pathogen inoculated DCA, CWG, LPS, and GB-treated seedlings was 2.55-, 2.74-, 2.85-, and 2.82-folds higher than that of the uninoculated samples, respectively.

### Hydroxyproline-rich glycoproteins (HRGPs) activity

**Figure 6:**
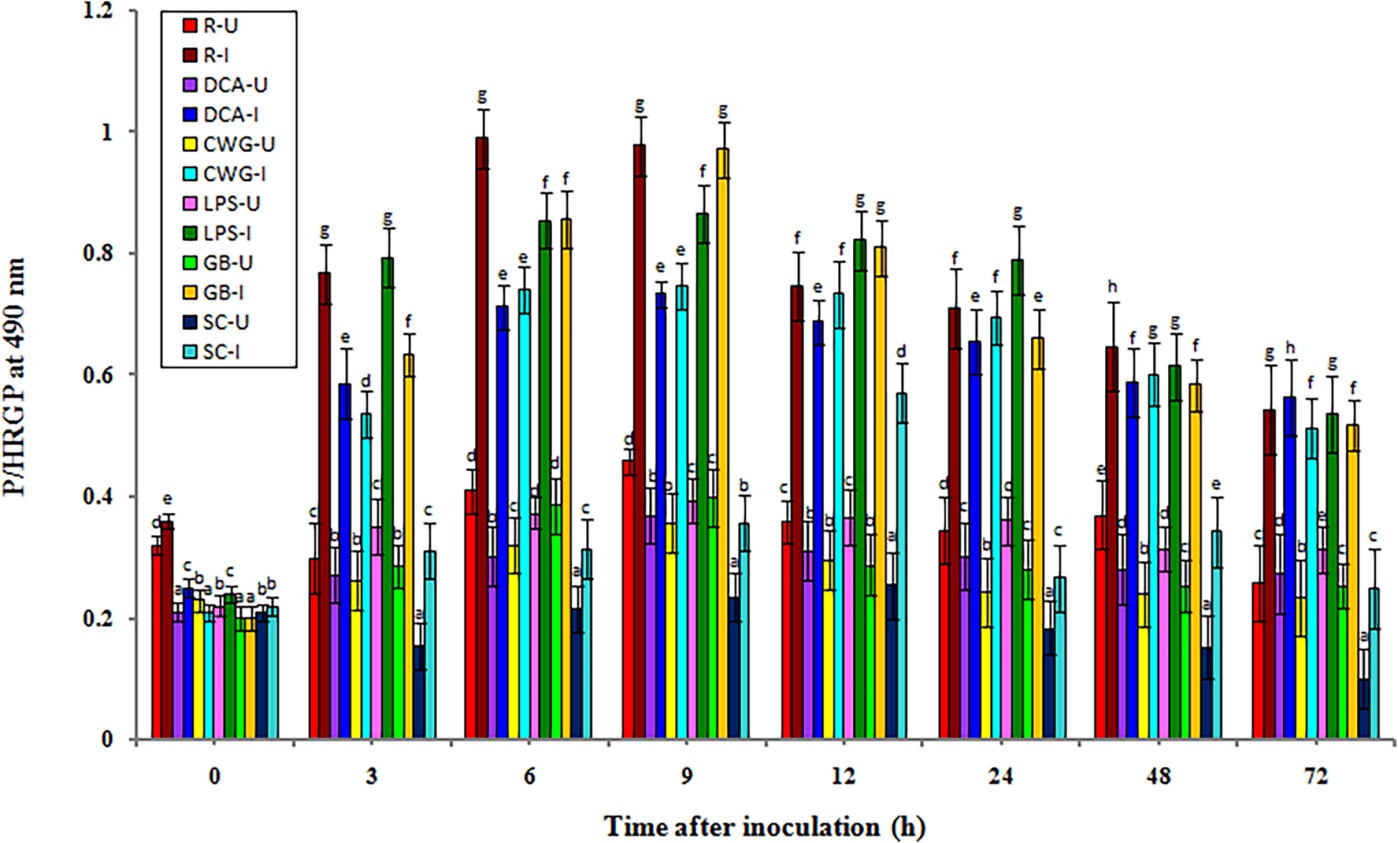
Temporal pattern of accumulation of HRGPs in 2-day old *P. glaucum* seedlings with (I-inoculated) or without (U-uninoculated) *Sclerospora graminicola* inoculation. R—resistant, S—susceptible, DCA—3,5-Dichloroanthranilic acid treated, CWG—Cell Wall Glucans isolated from the endophyte *Trichoderma hamatum* UOM 13, LPS—Lipopolysaccharides isolated from bacteria *Pseudomonas fluorescens* UOM 14, GB—Glycinebetaine an amino acid derivative. Bars indicate standard errors; means with different superscripts are significantly different, as shown by Tukey’s HSD test (*p* ≤ 0.05).

In general, the constitutive level of HRGP activity was recorded in all categories of seedlings with or without pathogen inoculation. In both pathogen inoculated and uninoculated samples, HRGP activity gradually increased from 3 h onwards and peaked at 9 h in elicitor treated, and resistant seedlings, whereas in control seedlings HRGP activity peaked at 24 h. HRGP activity was significantly higher in inoculated seedlings compared to the uninoculated seedlings in all treatments and at all tested time intervals (Fig. 6).

In pathogen inoculated seedlings HRGP activity peaked at 9 hpi and maximum HRGP activity was observed in GB-treated seedlings, which showed 0.971 HRGP activity which was on par with resistant seedlings which showed 0.9766 units HRGP activity. Among the elicitor treatments, at 9 hpi, GB-treated seedlings recorded 0.971 HRGP activity, followed by LPS, CWG, and DCA treatments which recorded 0.866, 0.746, and 0.733 units HRGP activity, respectively, whereas the control seedlings showed 0.356 units HRGP activity. HRGP activity at 9 hpi in GB, LPS, CWS, and DCA treated seedlings was 2.72-, 2.43-, 2.09-, and 2.06-folds higher than that of the control seedlings, respectively. Resistant seedlings showed 2.74-fold higher HRGP activity than the control seedlings. At 9 hpi GB-treated seedlings showed 1.12-, 1.3-, and 1.32-fold higher HRGP activity than LPS, CWG, and DCA treatments, respectively.

In seedlings without pathogen inoculation, the pattern of HRGP activity was similar to that of pathogen inoculated seedling but the level of activity was significantly lesser. Among the elicitor treatments at 9 h, GB-treated seedlings recorded maximum HRGP activity by showing 0.398 units activity followed by LPS, DCA, and CWG treatments which showed 0.393, 0.368, and 0.357 HRGP activity, respectively compared to the control seedlings which showed 0.235 units HRGP activity. GB treatment showed 1.69-folds higher activity than the susceptible control at 9 h. At 9 h HRGP activity in pathogen inoculated GB, LPS, CWG, and DCA treated seedlings was 2.44-, 2.20-, 2.01-, and 1.99-folds higher than that of the uninoculated samples, respectively.

### Quantitative real time PCR analysis (qPCR) for defense enzymes, hydroxyproline-rich glycoproteins, and pathogenesis-related proteins

#### PAL gene expression

**Figure 7:**
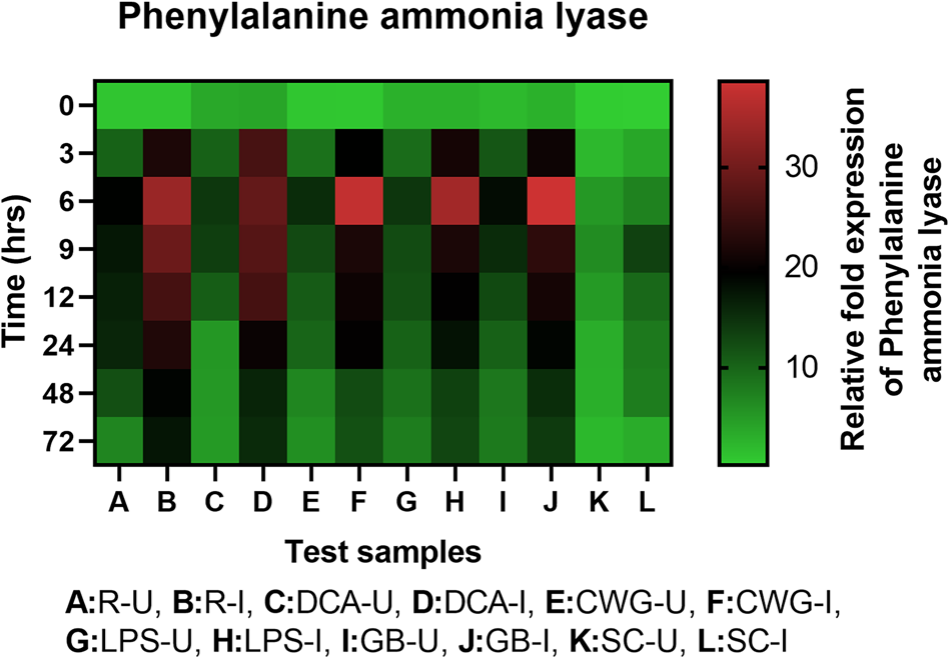
qRT-PCR determined relative expression of PAL genes in 2-day-old *P. glaucum* seedlings with (I) or without (U) *Sclerospora graminicola* inoculation harvested 0, 3, 6, 9, 12, 24, 48, and 72 h. R—resistant, S—susceptible, DCA—3,5-Dichloroanthranilic acid treated, CWG—Cell Wall Glucans isolated from the endophyte *Trichoderma hamatum* UOM 13, LPS—Lipopolysaccharides isolated from bacteria *Pseudomonas fluorescens* UOM 14, GB—Glycinebetaine an amino acid derivative. Expression levels were measured by qPCR and normalized to the constitutive PP2A gene. Values are means of experiments carried out in four replicates. The bars indicate ± SE and the data were analyzed by one-way ANOVA followed by Tukey's test and *p* ≤ 0.05.

Phenylalanine ammonia lyase transcripts was detected in all categories of seedlings with or without pathogen inoculation and the expression level was higher in resistant and elicitor treated seedlings compared to the susceptible controls. In all sets of seedlings PAL gene expression was higher in inoculated samples compared to the uninoculated samples at all time points (Fig. 7). Among the pathogen inoculated seedlings the expression of PAL gene was highest at 6 hpi in GB treated seedlings which was even higher than the resistant seedlings. Among the elicitor treated seedlings, at 6 hpi highest PAL gene expression was observed in GB treated seedlings followed by CWG, LPS and DCA treatments, which were 5.34-, 5.19-, 4.78- and 3.96-folds higher than that of the susceptible control seedlings, respectively. In seedlings without pathogen inoculation, pattern of PAL expression was similar to that of pathogen inoculated seedling but the level of expression was significantly lower. At 6 h, PAL gene expression in pathogen inoculated GB, CWG, LPS and DCA treated seedlings was 2.11-, 2.45-, 2.41-, and 2.02-folds higher than that of the uninoculated samples, respectively.

#### POX gene expression

**Figure 8:**
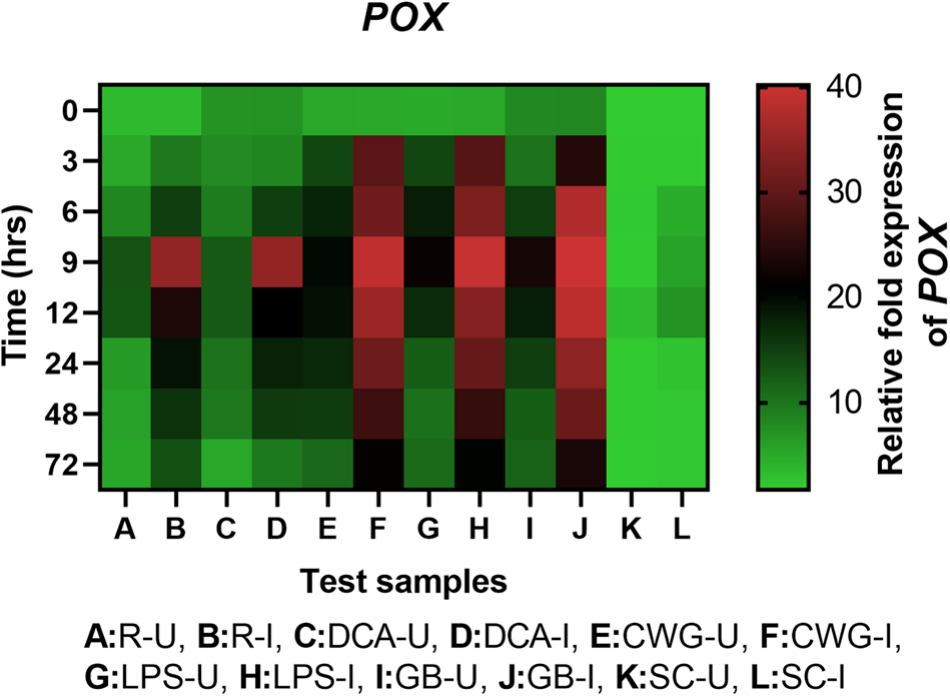
qRT-PCR determined relative expression of POX genes in 2-day-old *P. glaucum* seedlings with (I) or without (U) *Sclerospora graminicola* inoculation harvested 0, 3, 6, 9, 12, 24, 48, and 72 h. R—resistant, S—susceptible, DCA—3,5-Dichloroanthranilic acid treated, CWG—Cell Wall Glucans isolated from the endophyte *Trichoderma hamatum* UOM 13, LPS—Lipopolysaccharides isolated from bacteria *Pseudomonas fluorescens* UOM 14, GB—Glycinebetaine an amino acid derivative. Expression levels were measured by qPCR and normalized to the constitutive PP2A gene. Values are means of experiments carried out in four replicates. The bars indicate ± SE and the data were analyzed by one-way ANOVA followed by Tukey's test and *p* ≤ 0.05.

Peroxidase transcripts was detected in all categories of seedlings with or without pathogen inoculation and the expression level was higher elicitor treated seedlings compared to the resistant check and susceptible controls. In all sets of seedlings POX gene expression was higher in inoculated samples compared to the uninoculated samples at all time points (Fig. 8). Among the pathogen inoculated seedlings the expression of POX gene was highest at 9 hpi in elicitor treated seedlings which was even higher than the resistant seedlings. Among the elicitor treated seedlings, at 9 hpi highest POX gene expression was observed in GB treated seedlings followed by LPS, CWG and DCA treatments, which were 7.12-, 6.77-, 6.88- and 6.14-folds higher than that of the susceptible control seedlings, respectively.

In seedlings without pathogen inoculation, pattern of POX expression was similar to that of pathogen inoculated seedling but the level of expression was significantly lower. At 9 h, POX gene expression in pathogen inoculated GB, LPS, CWG, and DCA treated seedlings was 1.73-, 1.82-, 1.93-, and 2.75-folds higher than that of the uninoculated samples, respectively.

#### PPO gene expression

**Figure 9:**
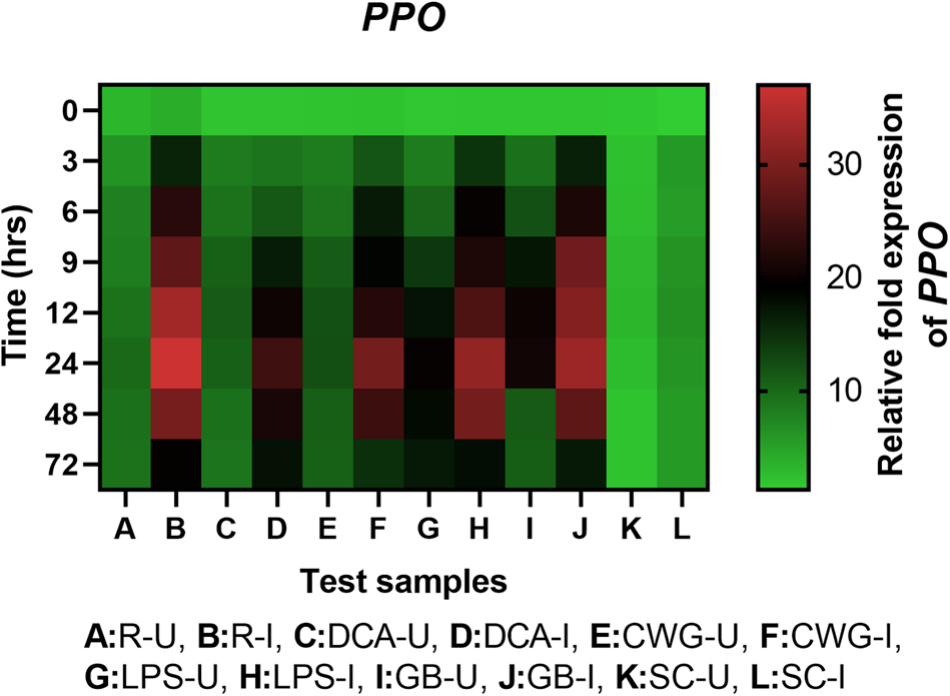
qRT-PCR determined relative expression of PPO genes in 2-day-old *P. glaucum* seedlings with (I) or without (U) *Sclerospora graminicola* inoculation harvested 0, 3, 6, 9, 12, 24, 48, and 72 h. R—resistant, S—susceptible, DCA—3,5-Dichloroanthranilic acid treated, CWG—Cell Wall Glucans isolated from the endophyte *Trichoderma hamatum* UOM 13, LPS—Lipopolysaccharides isolated from bacteria *Pseudomonas fluorescens* UOM 14, GB—Glycinebetaine an amino acid derivative. Expression levels were measured by qPCR and normalized to the constitutive PP2A gene. Values are means of experiments carried out in four replicates. The bars indicate ± SE and the data were analyzed by one-way ANOVA followed by Tukey's test and *p* ≤ 0.05.

Polyphenoloxidase transcripts was detected in all categories of seedlings with or without pathogen inoculation and the expression level was higher in resistant seedlings compared to the elicitor treated and susceptible controls. In all sets of seedlings PPO gene expression was higher in inoculated samples compared to the uninoculated samples at all time points (Fig. 9). Among the pathogen inoculated seedlings, at 24 hpi, resistant seedlings recorded highest PPO gene expression which was 6.08-folds higher than that of the control. And at 24 hpi, among the elicitor treated seedlings, highest PPO gene expression was observed in GB treated seedlings followed by LPS, CWG, and DCA treatments, which were 5.36-, 5.24-, 4.79- and 4.04-folds higher than that of the susceptible control seedlings, respectively.

In seedlings without pathogen inoculation, pattern of PPO expression was similar to that of pathogen inoculated seedling but the level of expression was significantly lesser. At 24 h, PPO gene expression in pathogen inoculated GB, LPS, CWG, and DCA treated seedlings was 1.58-, 1.62-, 2.38-, and 2.29-folds higher than that of the uninoculated samples, respectively.

#### β-1,3-glucanase gene expression

**Figure 10:**
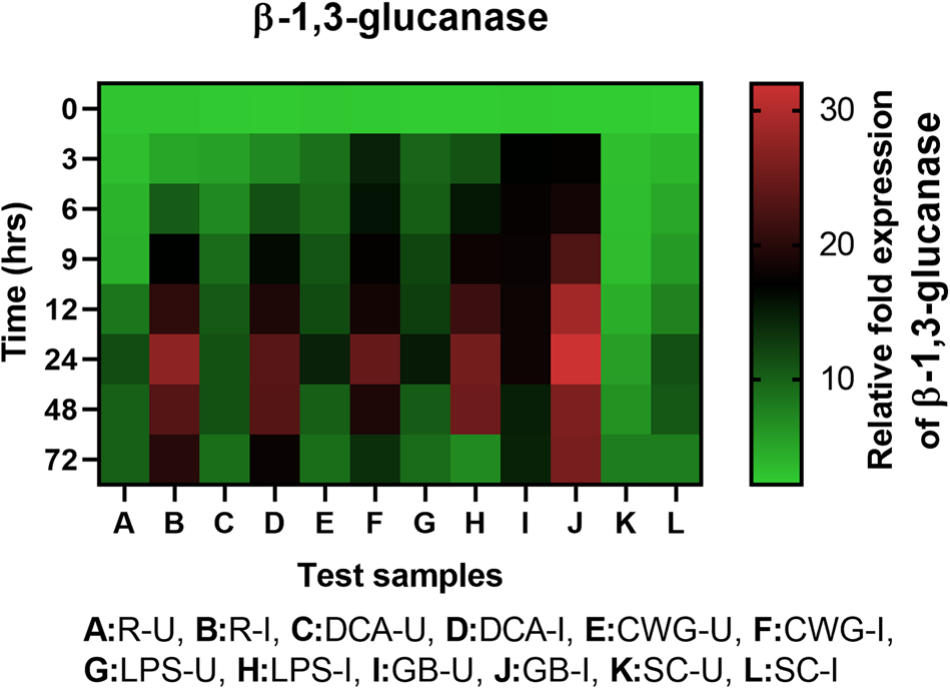
qRT-PCR determined relative expression of β-1,3-glucanase genes in 2-day-old *P. glaucum* seedlings with (I) or without (U) *Sclerospora graminicola* inoculation harvested 0, 3, 6, 9, 12, 24, 48, and 72 h. R—resistant, S—susceptible, DCA—3,5-Dichloroanthranilic acid treated, CWG—Cell Wall Glucans isolated from the endophyte *Trichoderma hamatum* UOM 13, LPS—Lipopolysaccharides isolated from bacteria *Pseudomonas fluorescens* UOM 14, GB—Glycinebetaine an amino acid derivative. Expression levels were measured by qPCR and normalized to the constitutive PP2A gene. Values are means of experiments carried out in four replicates The bars indicate ± SE and the data were analyzed by one-way ANOVA followed by Tukey's test and *p* ≤ 0.05.

β-1,3-glucanase transcripts was detected in all categories of seedlings with or without pathogen inoculation. In all sets of seedlings β-1,3-glucanase gene expression was higher in inoculated samples compared to the uninoculated samples at all time points (Fig. 10). Among the pathogen inoculated seedlings the expression of β-1,3-glucanase gene was highest at 24 hpi in all test samples. Maximum β-1,3-glucanase gene expression was observed in GB treated seedlings followed by LPS, CWG and DCA treatments. β-1,3-glucanase gene expression in GB, LPS, CWG and DCA treated seedlings was 2.85-, 2.27-, 2.17- and 2.09-folds higher than that of the control seedlings, respectively.

In seedlings without pathogen inoculation, pattern of β-1,3-glucanase expression was similar to that of pathogen inoculated seedling but the level of expression was significantly lesser. At 24 h, β-1,3-glucanase gene expression in pathogen inoculated GB, LPS, CWG and DCA treated seedlings was 1.76, 1.67, 1.66 and 2.10-folds higher than that of the uninoculated samples, respectively.

#### LOX gene expression

**Figure 11:**
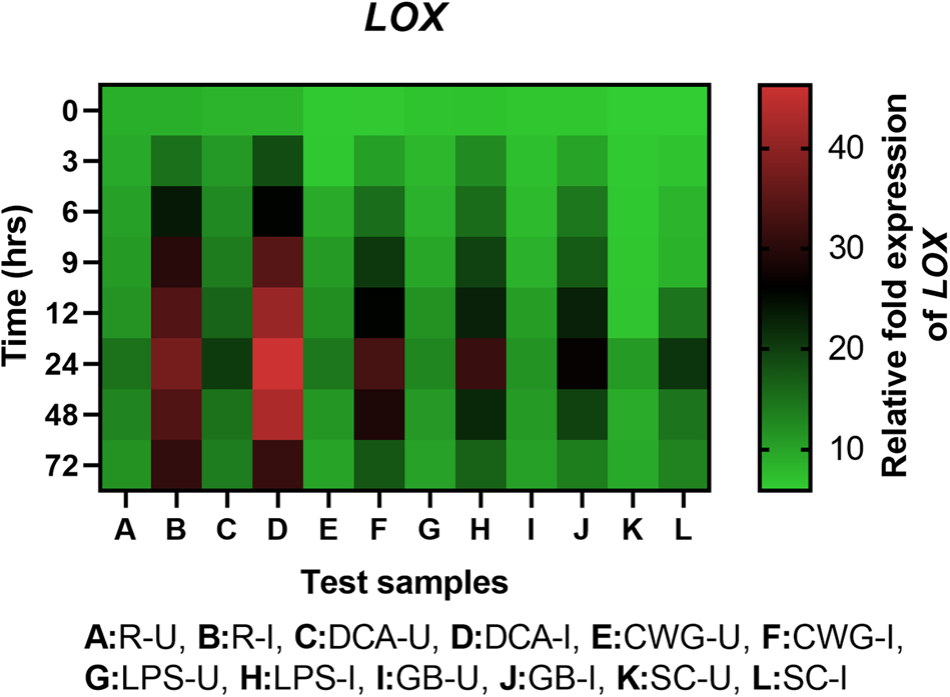
qRT-PCR determined relative expression of LOX genes in 2-day-old *P. glaucum* seedlings with (I) or without (U) *Sclerospora graminicola* inoculation harvested 0, 3, 6, 9, 12, 24, 48, and 72 h. R—resistant, S—susceptible, DCA—3,5-Dichloroanthranilic acid treated, CWG—Cell Wall Glucans isolated from the endophyte *Trichoderma hamatum* UOM 13, LPS—Lipopolysaccharides isolated from bacteria *Pseudomonas fluorescens* UOM 14, GB—Glycinebetaine an amino acid derivative. Expression levels were measured by qPCR and normalized to the constitutive PP2A gene. Values are means of experiments carried out in four replicates. The bars indicate ± SE and the data were analyzed by one-way ANOVA followed by Tukey's test and *p* ≤ 0.05.

Lipoxygenase transcripts was detected in all categories of seedlings with or without pathogen inoculation and the expression level was higher in resistant and elicitor seedlings compared to the susceptible controls at all time intervals. In all sets of seedlings LOX gene expression was higher in inoculated samples compared to the uninoculated samples at all time points (Fig. 11). Among the elicitor treatments, maximum LOX gene expression was observed in DCA treated seedlings followed by CWG, LPS and GB treatments. LOX gene expression in DCA, CWG, LPS and GB treated seedlings was 2.22-, 1.58-, 1.52- and 1.27-folds higher than that of the control seedlings, respectively.

In seedlings without pathogen inoculation, pattern of LOX expression was similar to that of pathogen inoculated seedling but the level of expression was significantly lesser. At 24 h, LOX gene expression in pathogen inoculated DCA, CWG, LPS and GB treated seedlings was 2.28-, 2.33-, 2.45- and 2.29-folds higher than that of the uninoculated samples, respectively.

#### HRGP gene expression

**Figure 12:**
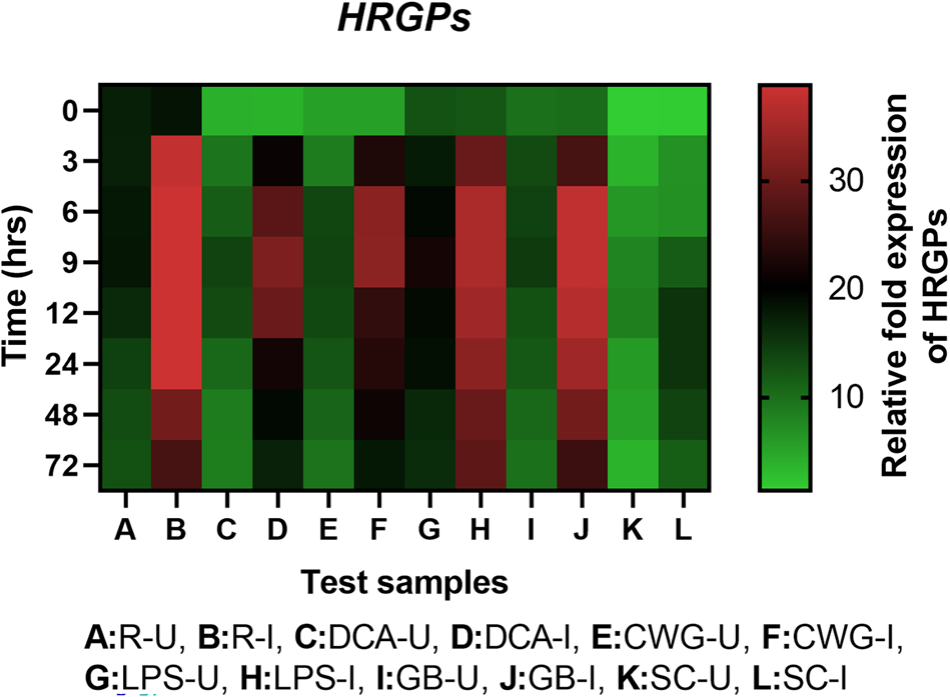
qRT-PCR determined relative expression of HRGPs genes in 2-day-old *P. glaucum* seedlings with (I) or without (U) *Sclerospora graminicola* inoculation harvested 0, 3, 6, 9, 12, 24, 48, and 72 h. R—resistant, S—susceptible, DCA—3,5-Dichloroanthranilic acid treated, CWG—Cell Wall Glucans isolated from the endophyte *Trichoderma hamatum* UOM 13, LPS—Lipopolysaccharides isolated from bacteria *Pseudomonas fluorescens* UOM 14, GB—Glycinebetaine an amino acid derivative. Expression levels were measured by qPCR and normalized to the constitutive PP2A gene. Values are means of experiments carried out in four replicates. The bars indicate ± SE and the data were analyzed by one-way ANOVA followed by Tukey's test and *p* ≤ 0.05.

Initially HRGP transcripts was detected in all categories of seedlings with or without pathogen inoculation and the expression level was significantly higher in resistant seedlings compared to the elicitor treated and susceptible controls. In all sets of seedlings HRGP gene expression was higher in inoculated samples compared to the uninoculated samples at all time points (Fig. 12). Among the pathogen inoculated, elicitor treated seedlings; at 9 hpi highest HRGP gene expression was observed in GB treated seedlings followed by LPS, CWG and DCA treatments, which were 3.24-, 3.06-, 2.82- and 2.71-folds higher than that of the susceptible control seedlings, respectively. HRGP gene expression in GB treated seedlings was 1.06-, 1.15-, and 1.19-folds higher than that of LPS, CWG, and DCA treated seedlings.

In seedlings without pathogen inoculation, pattern of HRGP expression was similar to that of pathogen inoculated seedling but the level of expression was significantly lesser, except for the LPS treatment. At 9 h, HRGP gene expression in pathogen inoculated GB, LPS, CWG, and DCA treated seedlings was 2.55-, 1.63-, 2.35-, and 2.26-folds higher than that of the uninoculated samples, respectively.

## Discussion

Preventing the pathogen ingress largely depends on the timely and appropriate perception of the pathogen by the host cells and subsequent activation of the defense mechanisms like production of secondary metabolites, reactive oxygen species (ROS), defense enzymes and pathogenesis-related proteins (PRs), all of which are coordinately expressed to stop the pathogen infection effectively^51^. By processes such as cell wall reinforcement, synthesis of antimicrobial metabolites, defense enzymes, PR proteins, and hypersensitive response, elicitor signaling efficiently primes the host defense responses and successfully controls pathogen manifestation^52^.

The findings of this study demonstrate that during elicitor-induced resistance to *P. glaucum* downy mildew disease. Glucanase, chitinase, PAL, POX, PPO, LOX, catalase, and HRGPs all play a vital role, and the speed and intensity with which these defensive enzymes and proteins are triggered and accumulated is strongly connected with the degree of resistance elicited by that particular elicitor. In general, all the tested elicitors, i.e., GB, LPS, CWG, and DCA showed earlier and higher activities of glucanase, PAL, POX, PPO, LOX and HRGPs compared to control.

The synthesis of various types of phenylpropanoid is highly dependent on the level of the PAL activity indicating that PAL is an important regulator of the phenylpropanoid pathway. Maximum PAL activity was observed in GB-treated seedlings, which was even significantly higher than resistant seedlings. Among the elicitor treatments, GB-treated seedlings had the highest PAL activity, followed by CWG, LPS, and DCA treatments, which were significantly higher than that of the susceptible controls. Our findings are consistent with those of previous research, which have shown induction, and elevation of PAL activities during induced systemic resistance against plant pathogens^17,19^. Furthermore, the involvement of PAL during the *P. glaucum* -*S. graminicola* interaction has been well described, implying that this enzyme plays a significant role in resistance^27,53^.

Peroxidases are implicated to play key role in plant–pathogen interaction and increased activities in POX have been thought to be key components in local and systemic disease resistance^54^. Defense enzymes, particularly peroxidases, prevent pathogen spread by forming polymerized phenolic barriers surrounding infection sites^55,56^ and triggering the production of anti-nutritive, antibiotic, and cytotoxic chemicals, resulting in increased pathogen resistance. In the present study, significantly higher POX activity was observed in inoculated seedlings compared to the uninoculated seedlings in all treatments and at all tested time intervals. In pathogen inoculated seedlings, among the elicitor treatments both LPS and GB treated seedlings recorded the highest POX activity followed by CWG, and DCA treatments which were significantly higher than control seedlings. Moreover, the role of POX during *P. glaucum* downy mildew interaction has been established earlier^57^. Systemic resistance to *P. glaucum* downy mildew disease has been effectively induced by an array of inducing agents such as *Aspergillus niger* (cell wall carbohydrates), Menadione sodium bisulphite (MSB), Ind-Ile-Me (1-oxo-indanoyl-l-isoleucine methyl ester), Chitosan, *Datura metel* extract, *Bacillus pumilus* INR7 and others; in all these studies, it was found that POX activity was significantly enhanced in *P. glaucum* seedlings corresponding with the increase in host resistance levels, implicating POX as an important marker of resistance in this system^28,35,58–61^.

The enzyme PPO oxidizes the less toxic phenolic compounds to highly toxic quinones and thereby plays a vital role in inhibiting the pathogens. PPO is also known to play a major role in lignin biosynthesis. In pathogen-inoculated seedlings PPO activity peaked at 24 hpi and maximum PPO activity was observed in GB-treated seedlings, which were even significantly higher than resistant seedlings. Among the elicitor treatments, GB-treated seedlings recorded the highest PPO followed by LPS, CWG, and DCA treatments.

Our results are in line with the earlier studies that have demonstrated enhanced activities of PAL, POX, and PPO due to different types of elicitor treatments, which have corresponded with the increased resistance to different pathogens in a variety of crop plants. Azoxystrobin and *P. fluorescens* both protected tomato plants by enhancing resistance against the late leaf blight disease caused by *Alternaria solani* and *Septoria lycopersici,* and the plants showed enhanced activities of POX, PAL, PPO and total phenols^62^. In lupin plants, systemic acquired resistance induction by different elicitors like *Pseudomonas fluorescens* and *Pseudomonas putida,* copper sulphate, indole butyric acid and potassium chloride was evaluated; it was observed that there was a substantial increase in the time course activities of different enzymes like PAL, POX, and PPO which resulted in enhanced resistance against *Fusarium* wilt disease caused by *Fusarium oxysporum* f. sp. *Lupine*^63^*. Trichoderma viride* treatment to black gram induced systemic resistance against *Fusarium oxysporum* wilt and *Alternaria alternata* blight infection, which was associated with a significant increase in the activities of the defense enzymes like POX, PPO, and PAL and total phenolic content^64^. Prior treatment of rice seeds with elicitor mixtures containing *Sargassum wightii*, *Pseudomonas fluorescens* and Annamalai mixture effectively triggered systemic resistance against sheath blight pathogen *Rhizoctonia solani* and such resistance induction was accompanied with maximum induction of POX, PPO, and PAL activities in the treated plants^65^.

β-1,3-glucanases are hydrolyzing enzymes that operate on the pathogen cell wall's β-1,3-glucans. Pathogens' cell walls can be degraded by glucanases, limiting their spread. These degraded fragments can also act as elicitors, triggering host defense responses. In the current study, β-1,3-glucanase activity was significantly higher in inoculated seedlings compared to the uninoculated seedlings in all the treatments and at all the tested time intervals. In pathogen inoculated seedlings, maximum β-1,3-glucanase activity was found in GB-treated seedlings, which was even significantly higher than resistant seedlings. Among the elicitor treatments, at 24 hpi, GB-treated seedlings recorded maximum β-1,3-glucanase activity, followed by LPS. Cell Wall Glucans and DCA treatments which were significantly higher than that of the control seedlings. Our findings back up previous reports of enhancement of glucanase activities during elicitor-induced resistance in many host–pathogen systems. Benzothiadiazole and SA which applied to rice plants as foliar spray developed resistance against *R. solani* infection, and it was associated with increased activities of defense enzymes like SOD, chitinase and β-1,3-glucanase, and phenols^66^. *T. harzianum*, *P. fluorescens*, *Ampelomyces quisqualis* treatment to cucumber plants showed higher levels of glucanase and chitinase, which resulted in induction of resistance against the downy mildew pathogen *Pseudoperonospora cubensis*^67^*.* Dalal et al.^68^ reported that indigenous endophytic microorganisms *Pseudomonas* sp., *Bacillus* sp., *Burkholderia* sp., *Streptomyces* sp., *Actinoplanes* sp., *Alternaria* sp., and *Fusarium* sp. induced systemic resistance against *R. solani* in soybean by significantly enhanced production of β-1,3-glucanase and chitinase. Two fungal elicitors derived from *Aspergillus flavus* and *A. parasiticus* induced various defenses responses,such as increased activities of β-1,3-glucanase, nitrate reductase (NR) and nitrite reductase (NiR), and total proteins, which led to increased resistance to *A. flavus* and *A. parasiticus*^69^*.* Prior treatment of rice seeds with elicitor mixtures containing *S. wightii*, *P. fluorescens* and Annamalai mixture effectively triggered systemic resistance against the sheath blight pathogen *R. solani* and showed enhanced β-1,3-glucanase activity^65^.The Damping-off disease of beet crop was effectively suppressed by induction of systemic resistance by the elicitor SA, which showed higher activities of β-1,3-glucanase and chitinase^70^.

Lipoxygenases directly attack the plant membrane system in a non-enzymatic manner and activate the membrane’s lipid peroxidation. The metabolites of lipid peroxidation include several enzymes of the LOX-dependent peroxidase pathway. Therefore, the LOX enzyme is also involved in the regulation of various defense responses like tissue necrosis, H_2_O_2_ accumulation, hypersensitive responses, etc. This LOX pathway leads to the synthesis of various compounds displaying antimicrobial or signaling activities or both. Stimulation of the LOX pathway results in the accumulation of hydroperoxides, which are important as the first line of chemical barriers against infection by pathogens. In the present investigation, LOX activity was significantly higher in inoculated seedlings compared to the uninoculated seedlings in all treatments and at all tested time intervals. In pathogen inoculated seedlings, maximum LOX activity was observed in DCA-treated seedlings, which was even significantly higher than resistant seedlings. Among the elicitor treatments, at 24 hpi, DCA-treated seedlings recorded the highest LOX activity, followed by CWG, LPS and GB treatments.

*Pseudomonas putida* strain BTP1 promoted induced systemic resistance in tomato against *Botrytis cinerea* by the higher accumulation of antifungal material and phytoalexins, along with the stimulation of LOX pathway^71^. *Colletotrichum capsici* derived cell wall elicitors induced systemic resistance against chilli anthracnose disease by the enhanced accumulation of hydrogen peroxide and increased activities of lipoxygenase^72^. Soaking of ginger rhizome seeds in SA and *Acalypha* leaf extract both induced systemic acquired resistance against *Pythium* infection, which is correlated with the increased activities of the defense enzyme LOX, however, SA induced more activities of these enzymes compared to *Acalypha* leaf extracts^73^. Bacterial spot disease in tomato caused by *Xanthomonas gardneri* (Xg) was effectively suppressed by different biotic elicitors like *Streptomyces setonii* (UFV618), *Bacillus cereus* (UFV592) and *Serratia marcescens* (UFV252), and an abiotic elicitor jasmonic acid (JA), which was correlated with induction of the increased activities of different defense enzymes including LOX^74^.

Hydroxyproline-rich glycoproteinsare structural components of plant cell walls which play a role in host defense responses to pathogen invasion. Infections with pathogens or treatments with pathogen-derived elicitors have boosted HRGP levels, resulting in resistance to a variety of diseases^13–15^. In the present study, HRGP activity was significantly higher in inoculated seedlings compared to the uninoculated seedlings in all the treatments and at all the tested time intervals. In pathogen inoculated seedlings, maximum HRGP activity was observed in the GB-treated seedlings, followed by LPS, CWG, and DCA treatments, respectively. Deposition and cross-linking of HRGPs in plant cell walls established effective resistance barriers against *S. graminicola* in *P. glaucum*^75^. Furthermore, treating susceptible *P. glaucum* seeds with various biotic and abiotic elicitors led to increased HRGP content in the cell walls, which was associated with enhanced resistance to downy mildew disease; in particular, seedlings raised from susceptible seeds treated *P. fluorescens* UOM SAR 14 showed the highest HRGP accumulation^76^.

It is established that during the process of induced resistance, host defense mechanism gets primed and de novo expression of defense-related genes get upregulated leading to their enhanced expression and accumulation in uninfected tissues, thereby protecting them against any future pathogen attack^77^. The findings of the present study clearly demonstrated that during elicitor induced resistance against *P. glaucum* downy mildew disease, several genes of the defense enzymes like glucanase, PAL, POX, PPO, LOX, and defense proteins like hydroxyproline-rich were upregulated, and the speed and intensity of their induction and overexpression positively correlated with the degree of resistance induced by that particular elicitor. In general, all the tested elicitors, i.e., GB, LPS, CWG and DCA showed earlier and higher mRNA transcript accumulation of glucanase, PAL, POX, PPO, LOX, and HRGP genes compared to the controls.

Our findings are in line with many other previous studies which have clearly demonstrated the role of PAL, POX and PPO gene expression during elicitor induced resistance against a wide range of pathogens in different crop plants^16,18,78–80^.

Various defense enzymes and signal molecules acting co-operatively may contribute to the development of an effective mechanical and chemical defense barrier in *P. glaucum* plants against *S. graminicola* invasion. This hypothesis is substantiated by our findings showing that high levels of POX, PPO, PAL, glucanase, LOX, and HRGP gene activities in elicitor treated *P. glaucum* seedlings which were correlated with high levels of resistance to downy mildew disease. Measurement of mRNA levels demonstrates that genes encoding POX, PAL, and HRGPs, were induced substantially after elicitor treatment and their expression became more prominent and pronounced after challenge with *Sclerospora graminicola*, which indicates that these elicitors are good priming agents. The enzyme products of the genes examined here are predicted to be involved in the biosynthesis of defense compounds, following pathogen inoculation.

In conclusion, the tested elicitors viz., GB, LPS, CWS and DCA were potential in eliciting the activities of the defense enzymes/proteinsand their genes, such as glucanase, PAL, POX, PPO, LOX and HRGP, however the speed and the extent of elicitation differed. The rate at which these enzyme activities and gene expression were triggered might be related to the aggressiveness of that particular elicitor in imparting resistance to the pathogen. The level of these enzymes and proteins increased distinctly after treatment of the elicitors themselves presenting a defense priming effect, and the pathogen challenge resulted in significantly higher levels and faster increments in enzyme and protein levels. Each elicitor showed variation in the types of enzymes that it stimulated and also in their levels and temporal pattern suggesting that the underlying defense mechanisms triggered and the response mounted by the host follows different patterns and pathways for each elicitor. Among different elicitors tested in this study, GB promoted an increase in the enzyme and protein activities in a more accentuated way and faster than the remaining elicitors, implicating that GB is a very promising elicitor for downy mildew disease resistance in *P. glaucum*. However, it is interesting to note that till now there are no reports on induction of PAL, POX, LOX, glucanase and HRGPs induction by GB. To the best of our knowledge, this is the first confirmed report on the use of GB elicitor for induction of downy mildew disease resistance in *P. glaucum*.

Taken together, our results have shown that GB, LPS, CWS and DCA elicitor-induced disease resistance in *P. glaucum* against *S. graminicola* assigns important roles for the defense enzymes glucanase, PAL, POX, PPO, LOX and the defense protein HRGPs. The earliness and intensity of expression of enzymatic activities determine the level of downy mildew resistance, which suggest to use them as markers for screening disease resistance elicitors.

## Supplementary Information

Supplementary Table 1.
