# Supplementary material for: Immunity elicitors for induced resistance against the downy mildew pathogen in pearl millet
Source: Sci Rep. 2022 Mar 8;12:4078. doi: 10.1038/s41598-022-07839-4 (PMC8904771; doi:10.1038/s41598-022-07839-4)
Supplement: Supplementary file 1 — Supplementary Table 1. [file 41598_2022_7839_MOESM1_ESM.docx]

**SUPPLEMENTARY TABLE**

**Table1:** Primer sequences used for qRT-PCR amplification of various genes.

| **Sl. No.** | **Target gene amplified** | **Forward primer sequence**  **(5’ to 3’)** | **Reverse primer sequence**  **(5’ to 3’)** |
| --- | --- | --- | --- |
| 1. | PAL | ATGGAGTGCGAGAACGGCC | CTGCGCGATGCTGAGGCT |
| 2. | POX | CCCCAGAAGCACATTTGTGA | CATGGCTGCGGGCGGAG |
| 3. | PPO | AGTCGAGGTTTGGCCACCAT | CCACCTGATGCGCTCGATG |
| 4. | β-1,3-glucanase | AGCATTCGCAGCCATTCCTA | TGCATGCACGGATTATGGGT |
| 5. | LOX | GCTGCTGATCGAGGACTACC | GACGCGATCCAGATGATGGT |
| 6. | HRGP | GCCTAAGCCGAAGCCACCAA | GCGTGTAGGTCGGAGGAGTT |
| Reference housekeeping gene | | | |
| 1. | PP2A | TGAGAGCAGACAAATCACTCAA | AAGAGCTGTGAGAGGCAAATAA |
